# Nuclear Dynamics and Chromatin Structure: Implications for Pancreatic Cancer

**DOI:** 10.3390/cells10102624

**Published:** 2021-10-01

**Authors:** Luis F. Flores, Brooke R. Tader, Ezequiel J. Tolosa, Ashley N. Sigafoos, David L. Marks, Martin E. Fernandez-Zapico

**Affiliations:** Schulze Center for Novel Therapeutics, Division of Oncology Research, Mayo Clinic, Rochester, MN 55905, USA; flores.luis@mayo.edu (L.F.F.); tader.brooke@mayo.edu (B.R.T.); tolosa.ezequiel@mayo.edu (E.J.T.); Sigafoos.Ashley@mayo.edu (A.N.S.); marks.david@mayo.edu (D.L.M.)

**Keywords:** nuclear morphology, chromatin, gene expression, pancreatic cancer, nuclear lamina

## Abstract

Changes in nuclear shape have been extensively associated with the dynamics and functionality of cancer cells. In most normal cells, nuclei have a regular ellipsoid shape and minimal variation in nuclear size; however, an irregular nuclear contour and abnormal nuclear size is often observed in cancer, including pancreatic cancer. Furthermore, alterations in nuclear morphology have become the ‘gold standard’ for tumor staging and grading. Beyond the utility of altered nuclear morphology as a diagnostic tool in cancer, the implications of altered nuclear structure for the biology and behavior of cancer cells are profound as changes in nuclear morphology could impact cellular responses to physical strain, adaptation during migration, chromatin organization, and gene expression. Here, we aim to highlight and discuss the factors that regulate nuclear dynamics and their implications for pancreatic cancer biology.

## 1. Introduction

For over a century, scientists have reported that normal nuclear morphology is disrupted in disease states, including cancer. Sir Lionel Beale first noticed this phenomenon in the sputum of a cancer patient in 1860 [1]. Not quite a century later, George Papanicolaou developed a diagnostic test for cervical cancer, the Pap smear, that is still used today to identify abnormal nuclear morphology in samples taken from the cervix [2,3]. These cancer-associated alterations in nuclear morphology include changes in size, loss of ellipticity, presence of invaginations or blebs, and changes in nuclear texture (i.e., dark and light areas). The contents of the nucleus are arranged in a non-random fashion through higher-order chromatin structures [4,5,6]. These chromatin structures play a role in determining the morphology of the nucleus, and increasing evidence demonstrates that chromatin organization is perturbed in cancer cells compared to their normal counterparts. The changes in nuclear morphology seen in cancer cells are also associated with changes in cellular functions, including gene expression and cytoskeletal dynamics; however mechanistic links between aberrant nuclear morphology, altered chromatin organization, and the transformation of cancer cells have yet to be fully elucidated. In this review, we first outline the key components of the nuclear envelope and concepts of chromatin organization. We then review how these factors regulate nuclear dynamics, with a focus on their dysregulation in pancreatic cancer, especially pancreatic ductal adenocarcinoma (PDAC), the most common histological subtype of this dismal malignancy. This review focuses on nuclear morphology and chromatin organization in cancer. We mainly discuss studies performed in mammalian cells and systems as many fine reviews have covered related topics in nonmammalian systems [7,8,9,10,11,12,13,14,15]. Finally, we discuss unanswered questions concerning the interplay between nuclear dynamics and chromatin structure and the impact of these processes on pancreatic cancer.

## 2. Nuclear Structure

### 2.1. The Nuclear Envelope

**Figure 1:**
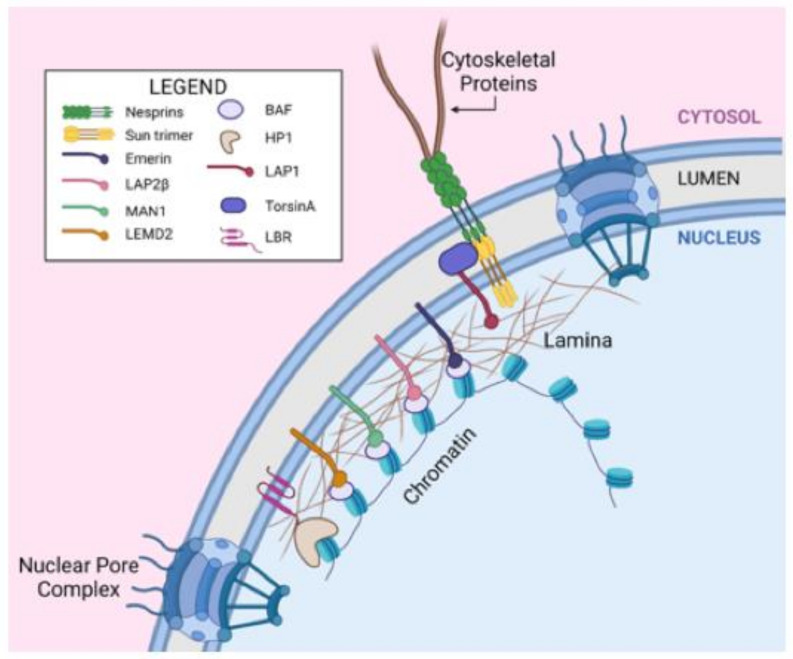
Components of the nucleus and the nuclear envelope involved in nuclear dynamics. Nesprin proteins are a component of the outer nuclear membrane (ONM) that interact with various cytoskeletal proteins in the cytosol. SUN proteins are components of the inner nuclear membrane (INM) and can interact with nesprin proteins to form the Linker of Nucleoskeleton and Cytoskeleton (LINC) complex, which is a structural and mechanical feature of the nuclear envelope. TorsinA is contained in the nuclear envelope lumen and can interact with LINC complexes and the INM protein LAP1. Proteins from the LEM domain family (LAP2β, Emerin, MAN1, LEMD2) are INM proteins that interact with Barrier to Autointegration Factor (BAF) to facilitate heterochromatin anchoring to the lamina. The nuclear lamina is a meshwork of intermediate filaments (A- and B-type lamins) that sits inside the INM and gives structural support to the nucleus. Lamin B Receptor (LBR) is another INM protein that can interact with heterochromatin through the linker protein HP1. Nuclear Pore Complexes span both nuclear membranes to provide transport between the nucleus and cytosol. Created with biorender.com (accessed on 1 September 2021).

The nucleus is surrounded by the nuclear envelope, which is composed of two concentric bilayer membranes, an outer nuclear membrane (ONM) and an inner nuclear membrane (INM), which each possess a specialized set of proteins (Figure 1) [16,17]. These two membrane layers are separated by the nuclear envelope lumen, a space of 30–50nm. Large multiprotein structures, the nuclear pore complexes, span the nuclear envelope. Just interior to the INM is the nuclear lamina, a dense protein meshwork consisting of lamins and associated proteins that plays key roles in tethering heterochromatin to the nuclear periphery [18,19].

The ONM is continuous with the endoplasmic reticulum (ER) and retains many proteins in common with the ER [20,21]. Other proteins reside primarily in the ONM, including nesprins, key transmembrane ONM proteins involved in nuclear mechanosensing [22]. The cytoplasmic N-terminal region of nesprins 1-4 contains spectrin repeats and other nesprin isoform-specific domains, allowing individual nesprins to interact with actin filaments, microtubules, and/or intermediate filaments [22,23,24,25]. The C-termini of nesprins possess KASH (Klarsicht, ANC-1, or Syne Homology) domains that interact with the C-termini of SUN proteins within the nuclear envelope lumen (Figure 1). SUN domain proteins (SUN1 and SUN2) form homo- or heterotrimers in the INM [26]. The N-termini of SUN proteins traverse the INM and interact with nuclear lamina proteins [23,27]. Nesprins and SUN proteins together make up the linker of nucleoskeleton and cytoskeleton (LINC) complexes, which transmit plasma membrane mechanical information to the nucleus [28].

The INM is known to contain >60 proteins, only some of which are characterized [16,21]. One important group of INM proteins is the LEM family, which contain the LAP2-Emerin-MAN1 (LEM) domain, a region that binds to the Barrier to Autointegration Factor (BANF1) (Figure 1) [18,21,29]. Mammals have seven genes encoding LEM proteins, including Emerin, LAP2 (gene name *TMPO*, encodes multiple splice forms including LAP2α and LAP2β), MAN1, LEMD1, LEMD2, ANKLE1, and ANKLE2 [30,31,32,33,34]. Although not all LEM protein functions are well-characterized, each of these proteins appear to be involved in nuclear membrane architecture or maintenance, and/or mitotic processes. Most importantly for our discussion, LEM proteins are well-known regulators of nuclear architecture, via tethering heterochromatin to the nuclear periphery either directly or indirectly through BANF1 [18,21,35,36]. Not all mammalian cells and tissues contain the same assortment of LEM proteins, and their functions are thought to be distinct, although some may be partially redundant [31,37]. Most LEM proteins are anchored to membranes via either one (Emerin, LAP2β, LEMD1, ANKLE2) or two (LEMD2, MAN1) transmembrane domains; however, ANKLE1 and LAP2α lack transmembrane domains [21,30,38,39].

Emerin, LAP2β, MAN1, and LEMD2 are primarily localized to the INM [31,40,41]. Possibly the most well-characterized LEM protein is Emerin, which was identified in 1994 as the locus mutated in X-linked recessive Emery–Dreifuss muscular dystrophy [31]. Emerin has been shown to be involved in nuclear lamina organization via its interaction with A-type lamins, chromatin organization via BANF, and cytoskeletal/nuclear signaling via interaction with nesprins [31,42]. In addition, Emerin recently has been shown to interact with tubulin at the mitotic spindle and play a role in nuclear reassembly after mitosis [43]. LAP2 encodes several splice forms, with LAP2α and LAP2β being the most abundant forms [44,45,46,47]. While LAP2β has a transmembrane domain and resides in the INM, LAP2α has been demonstrated to bind a pool of nucleoplasmic lamin A/C [44]. In addition to binding chromatin via BANF, LAP2β is reported to act as a transcriptional repressor via its interaction with HDAC3, and can act as an intranuclear reservoir for certain transcription factors, such as GLI1 [47,48,49,50]. MANI has been shown to antagonize TGFβ signaling by binding SMAD2/3 [51]. A recent protein interactome study suggests that MAN1 may also play a role in ribonucleoprotein complex assembly [52]. LEMD2 has recently been shown to play a role in nuclear membrane closure after mitosis via interaction with CHMP7 and other ESCRT factors [53,54].

LEMD1, ANKLE1, and ANKLE2 are less well-characterized and are not localized mainly to the nuclear lamina [38,55]. LEMD1 was identified as a testes-associated gene but has since been found to be overexpressed in a number of malignancies, including colon, thyroid, and prostate cancer, and oral squamous cell carcinoma [56,57,58,59]. One study has expressed epitope-tagged LEMD1 and shown its localization to be apparently extranuclear, possibly associated with the endoplasmic reticulum [39]. Depletion of LEMD1 in various cancer cell types decreased cell growth, invasiveness, and epithelial–mesenchymal transition [57,58,59]; however, a specific cellular function for LEMD1 has yet to be determined. ANKLE1 is a nonmembrane protein normally located mainly in the cytosol, but able to be actively imported into and exported from the nucleus [38,60]. Unique among LEM proteins, ANKLE1 possesses a C-terminal endonuclease domain [60]. Recent studies indicate that ANKLE1 may function in the removal of branched DNA at the end of mitosis [61,62]. Finally, ANKLE2 has been reported to be localized to the ER in human cells but to the nuclear envelope in *C. elegans* [39,55]. ANKLE2 controls postmitotic nuclear envelope formation by regulating the dephosphorylation of BANF [55,63]. ANKLE2 is reported to be overexpressed in breast cancer, and its expression has been shown to positively regulate growth in breast cancer cells [64]. Cellular depletion of ANKLE2 results in the formation of abnormal lobulated nuclei [55,65], similar to effects seen with the knockdown of other LEM proteins [66,67] (see Table 1).

Another important INM protein associated with the nuclear lamina is Lamin B receptor (LBR), which binds chromatin via HP1 proteins (HP1α and HP1β), chromatin crosslinkers that bind trimethylated histone 3, lysine 9 (H3K9me3) [19,125]. LBR also is reported to directly bind H4K20me2 and the heterochromatic methyl binding protein, MeCP2 [126,127]. LAP1 (gene name *TOR1AIP1*) is an integral membrane protein that interacts with lamins, chromatin, Emerin, and Torsins, AAA+ ATPases of the nuclear membrane lumen that are involved in nuclear pore complex biogenesis, nucleo-cytoskeletal coupling, and protein quality control [20,86,128,129]. PRR14 is a recently discovered lamina-associated protein that also binds HP1 and requires Lamin A/C for its nuclear lamina association [130,131]. A series of recent studies by the Rebelo group have identified a potential role for LAP1 in DNA damage repair [132]. These authors previously found that LAP1 is dephosphorylated by PP1 [133,134], but the significance of LAP1 phosphorylation was unknown. More recent studies showed that, upon treatment of cells with DNA damaging agents such as bleomycin or H_2_O_2_, LAP1 physically associates with the shelterin complex subunit, TRF2, with partial intracellular localization of this complex with DNA damage repair markers (e.g., γ-H2AX) [132,135]. TRF2 was demonstrated to interact with the phosphorylated form of LAP, identifying a novel mechanism of LAP1 regulation [132]. The authors speculated that LAP1 may be phosphorylated by ATM/ATX kinases during DNA damage [132].

The structural proteins of the nuclear lamina are lamins. Lamins are type V intermediate filament proteins that self-assemble into 3.5 nm thick tetrameric filaments [136,137] and are the products of three genes: *LMNA*, which encodes Lamin A and Lamin C via alternative splicing, and *LMNB1* and *LMNB2*, which encode Lamin B1 and Lamin B2, respectively [138]. Lamins A and C are thought to be retained at the INM via their interactions with INM transmembrane proteins, whereas B-type lamins are held in place via their farnesylated C-termini and their interaction with LBR, an INM protein [136,138]. In addition to its presence in the nuclear lamina, a small fraction of Lamin A has been shown to occur in the nucleoplasm, in association with LAP1α and euchromatin [35,139,140]. All mammalian cell types possess B-type lamins, whereas A-type lamins are mainly expressed in differentiated cells [141]. Studies indicate that A-type and B-type lamins form distinct but overlapping networks in the nuclear lamina [142,143].

### 2.2. Chromatin Organization in the Nucleus

**Figure 2:**
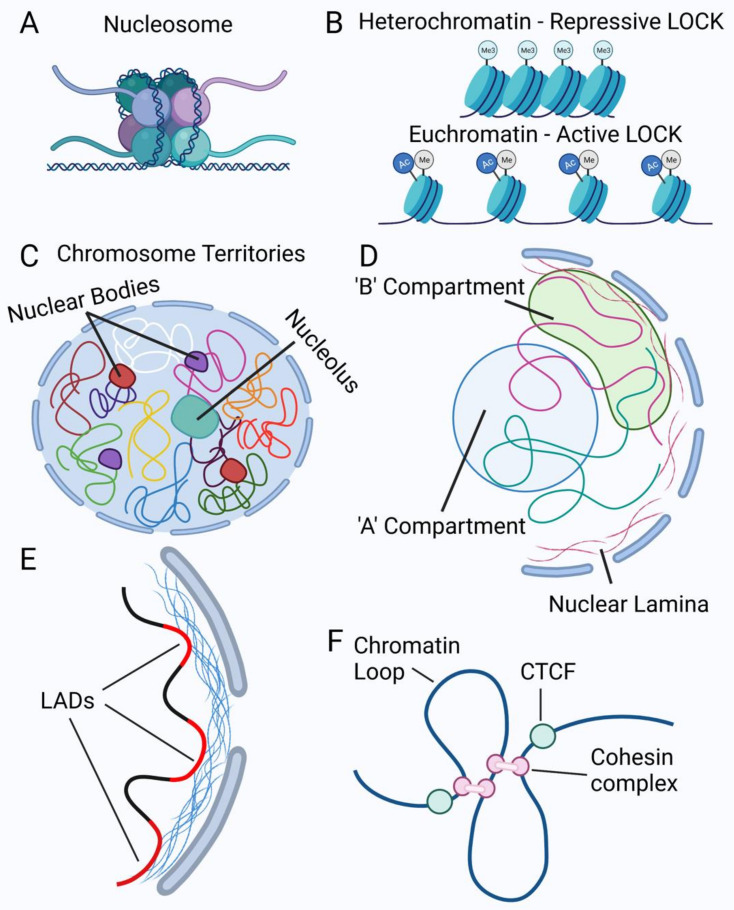
Chromatin organization in the nucleus. (**A**) Example of a nucleosome showing DNA wrapped around a histone octamer consisting of two H2A and H2B dimers, two H3 dimers, and two H4 dimers. (**B**) Example of heterochromatin or closed chromatin (top) and euchromatin or open chromatin (bottom). Long stretches of histones with similar lysine modifications illustrate Large Organized Chromatin Lysine (‘K’) modifications (LOCKs). The closed or open state refers to the accessibility of the chromatin to transcription factors or other DNA binding proteins. (**C**) Representation of chromosome territories where each colored line depicts one chromosome within the nucleus. Each chromosome is shown occupying its own space within the nucleus. The nucleolus is depicted here interacting with parts of some chromosomes. Nuclear bodies are also present, with examples of Cajal bodies (purple) and PML bodies (red) being shown. (**D**) Depiction of active ‘A’ compartment in the blue circle, indicating more open chromatin and actively transcribed genes, and ‘B’ compartment, which is mainly heterochromatin and therefore transcriptionally inactive. (**E**) Examples of Lamina-Associated Domains (LADs), shown here as red chromatin regions. (**F**) Examples of chromatin loops formed by cohesin complexes and demarcated by CTCF proteins. Created with biorender.com (accessed on 1 September 2021).

The nuclear lumen contains chromatin, which is the genomic DNA in association with RNAs, histones, and other chromatin proteins. At the broadest level, nuclear chromatin is divided into heterochromatin and euchromatin, terms that were coined based on areas of densely packed chromatin (heterochromatin) vs. less dense chromatin (euchromatin) visualized in the nucleus by staining (e.g., DAPI or Hoechst) or by electron microscopy [144,145] (Figure 2B). In a simplified conceptual model, heterochromatin represents silenced regions of the genome, including repetitive sequences and repressed genes. Genes that are facultatively repressed, i.e., activated in only in some tissues or by signaling events, are also found in heterochromatin. In contrast, euchromatin contains genes that are actively being expressed. Morphologically, euchromatin is generally located in the nuclear interior, whereas heterochromatin is mainly present at the nuclear periphery associated with the nuclear lamina. Heterochromatin is also present within the nucleus associated with nucleoli and pericentromeric chromatin [5,146,147,148].

The smallest level of chromatin structure is the nucleosome, an octameric complex consisting of two molecules each of histones H2A, H2B, H3, and H4, around which ~146 base pairs of double-stranded genomic DNA are wound [149,150,151] (Figure 2A). Nucleosomes are connected by linker regions of 10–80 bp DNA associated with histone H1 [152]. Canonical histones can be substituted by histone variants during specialized activities such as transcription, DNA repair, or cell division [153,154,155]. Nucleosomes help to stabilize DNA and are involved in maintaining genomic DNA at various levels of compaction and regulating transcription by limiting the access of macromolecules to DNA [151,155]. A key regulatory element of nucleosomal histones is that their C-terminal tails may be post-translationally modified. These modifications, such as methylations (me) and acetylations (Ac), have been demonstrated to be associated with gene activation (e.g., H3K4me1, H3K4me3, H3K27Ac) or gene repression (H3K9me3, H3K27me3) and are referred to as the histone code [156,157,158]. The DNA in chromatin can also be modified by methylation. DNA sequences enriched in cytidine followed by guanidine (CpGs) may be methylated or demethylated in processes that impact gene expression and chromatin organization [159,160].

Interphase mammalian cells possess genomic DNA with a total length of ~2 m (all chromosomes combined) [161]. The ability to pack this chromatin into a single nucleus while retaining functionality requires an exceptional level of organization. With the advent of new technologies, recent investigations have characterized nuclear chromatin at different levels of three-dimensional (3D) organization, including chromosome territories, A/B compartments, topologically associating domains, lamina-associated domains, large organized chromatin (modified) histone lysine blocks, and chromatin loop domains (Figure 2) [145,162,163,164].

The term chromosome territory (CT) was coined by Boveri, who, upon microscopic studies of horse roundworms, described that individual chromosomes visible in mitosis occupied distinct territories even in interphase (Figure 2C) [165,166]. Fluorescence in situ hybridization techniques have confirmed the existence of spatially distinct chromosome territories [167]. While the positions of individual chromosomes are not tightly fixed, each chromosome is reported to exhibit a somewhat consistent radial distance from the nuclear center. Further studies have illustrated that gene-poor chromosomes tend to be closer to the nuclear periphery and gene-rich chromosomes closer to the nuclear center, and that, within CTs, repressed genes are often at the nuclear periphery while activated genes are generally present closer to the nuclear center [17,168,169]. A refinement of the CT concept describes the presence of an interchromatin compartment, a series of channels between and pervading the CTs [163]. These channels allow the movement of protein and RNA machinery for transcription, splicing, and DNA repair to appropriate chromatin domains, and they are regions of high transcriptional activity.

The development of chromosome conformation capture techniques (Hi-C and related methods) has allowed the exploration of the physical proximities between different regions of the genome. These techniques are based on the ability to crosslink DNA regions that are near each other, and then analyze the sequences captured by high-throughput methods. Using these methods, overall genome DNA co-interactions can be mapped. Initial studies have described DNA interactions as occurring in two compartments, with transcriptionally active regions tending to associate with each other in the ‘A’ (‘active’) compartment, and transcriptionally inactive regions in the ‘B’ compartment [6] (Figure 2D). Compartmentation of chromatin may facilitate enhancer–promoter interactions, allow transcription factors to be proximal to multiple active genes, and keep inactive regions close together to maintain heterochromatin boundaries [6,145,170]. The A and B compartments, defined by DNA proximity, can be seen as similar to the euchromatin/heterochromatin divisions as defined visually and biochemically [171]. Using higher-level resolution with chromatin conformation capture techniques, DNA regions can be grouped into topologically associated domains (TADs) [172,173,174]. TADs are recognized regions within single chromosomes or adjacent areas of multiple chromosomes that have close DNA interactions. The boundaries of TADs are typically demarcated by CTCF proteins, active promoters, transcriptional start sites, housekeeping genes, and repetitive elements (Alu/B1 and B2 SINE retrotransposons in mice and Alu SINE elements in humans) [4]. Chromatin loop domains (Figure 2F) formed through interactions between cohesin and DNA are thought to be components of TADs [175,176].

Lamina-associated domains (LADs) were first defined in *Drosophila* and human lung fibroblasts using DNA adenine methyltransferase (DAM-ID)–Lamin B1 fusion proteins to globally methylate adenines in DNA regions proximal to Lamin B1 in living cells [177,178] (Figure 2E). DAM-ID has continued to be used to define LADs in other systems and additional studies have investigated the organization of LADs using chromatin immunoprecipitation sequencing (ChIP-seq) [50,179,180,181]. LADs consist predominantly of transcriptionally inactive DNA that is localized to the nuclear periphery [5]. LADs are typically marked by H3K9me2 or 3 and are characterized by having repetitive GAGA motifs [5]. Several studies have shown that constitutively present LADs include many repetitive elements, including LINEs and LTRs, and that LAD regions, as defined by DAM-ID-Lamin B1, overlap with pericentromeric and perinucleolar chromatin [50,177,182,183]. Similar in concept to LADs are large organized chromatin (modified) histone lysine blocks (LOCKs), blocks of chromatin marked with post-translationally modified histone lysines as identified by ChIP-chip DNA arrays or ChIP-seq for particular histone marks (Figure 2B). The term LOCKs was first used to represent H3K9me2 domains [184], but has since been used for chromatin blocks marked by additional repressive or activating histone marks [160,185,186].

### 2.3. Nuclear Mechanics

The mechanical properties of the nucleus have been reported to be altered in cancer cells [16]. Several studies have suggested that nuclear softening increases the invasiveness of tumor cells [16,187,188], and a recent study found that cancer cell nuclei soften during migration through an endothelial cell layer [189]. Nonetheless, the stiffness of PDAC cells has been shown to positively correlate with invasiveness [190]. Nuclear stiffness can be regulated by several factors, including the extracellular matrix, cytoskeleton, the nuclear lamina, and chromatin organization.

Cytoskeletal filaments can communicate cellular and extracellular stiffness with nuclei via the LINC complex, composed of nesprins in the ONM and SUN proteins in the INM. Nesprin-1 and -2 connect to actin filaments, Nesprin-1α and Nesprin-4 bind to microtubules via kinesin, and Nesprin-3 binds to Plectin, allowing the potential for Nesprins to indirectly interact with all cytoskeletal networks [191,192,193,194,195]. The C-terminal SUN domains of the SUN proteins interact with the C-terminal domains of Nesprin proteins [196], while the SUN protein N-termini cross the INM, allowing the transmittal of mechanosignals to the nuclear lamina and nuclear pore complex, culminating in changes in chromatin organization and gene expression [17,22,197]. Although SUN proteins were thought to be primarily redundant in function, a recent study found that SUN2 promoted the activation of RhoA, a key regulator of the actin cytoskeleton, whereas SUN1 acted in competition with SUN2 to inhibit RhoA activity [198]. The microtubule cytoskeleton can also communicate with the nucleus independently of the LINC complex via interactions between the microtubule motors, Kinesin and Dynein, and nuclear pore complex proteins [199,200].

An elegant study by Guilluy et al. used isolated HeLa cell nuclei to investigate the function of the LINC complex [201]. Isolated nuclei were presented with magnetic beads coated with anti-Nesprin-1 antibodies; the beads were manipulated with magnetic tweezers and bead displacement recorded as a measure of stiffness. When nuclei were challenged by repeated pulses of force by the manipulation of the beads, the nuclei became stiffer. Treatment of isolated nuclei with latrunculin A or cytochalasin D did not alter the ability of nuclei to stiffen, suggesting that intranuclear actin polymers were not involved in the stiffening process. Similarly, trichostatin (TSA) treatment to cause the decondensation of chromatin had no impact on the stiffening response. However, RNAi-based depletion of Lamin A/C prior to nuclear isolation induced the softening of nuclei and an inability to respond to force by stiffening. Interestingly, Emerin depletion caused nuclear stiffening but inhibited the nuclear stiffening response to force. This study demonstrates that nuclei have an intrinsic ability to respond to stimulation via the LINC complex and supports the roles of Lamin A/C and Emerin in regulating nuclear stiffness.

An investigation by Stephens et al. used nuclei from Vimentin knockout mouse embryonic fibroblasts (MEFs) to probe the role of chromatin state in nuclear stiffness using a two-micropipet system to estimate stiffness [202]. In this study, pretreatment of cells before nuclear isolation with the histone deacetylase inhibitors, valproic acid, or trichostatin, to increase euchromatin, or with the EZH2 inhibitor 3-deazaneplanocin-A (DZNep) to decrease heterochromatin, each decreased nuclear stiffness. A third study used MEFs with triple knockout of Lamins A/C, B1, and B2, and rescue of lamin expression using lentiviral infection [203]. Nuclear stiffness was measured in intact cells by micropipet aspiration, i.e., deformation of the nucleus into a micropipet tip upon the application of suction [203]. This study demonstrated that both Lamin A/C and Lamin B1 contributed to nuclear stiffness, and that decondensation of chromatin with TSA only increased nuclear softness when Lamins were absent. Together, these three studies provide significant evidence that intrinsic nuclear stiffness is regulated by the LINC complex, Lamins, and, secondarily, by chromatin state.

## 3. Mechanisms of Alterations in Nuclear Morphology

There are many potential mechanisms that could lead to changes in nuclear shape, size, or physical properties such as stiffness or flexibility. Perhaps the best known proteins that affect nuclear morphology are Lamin A, Emerin, and other lamina-associated (MAN1, LAP1, LAP2, LBR, LEM2) and LINC complex (Nesprins 1 and 2, SUN1) proteins whose loss or mutation cause laminopathies or nuclear envelopathies, a series of genetic conditions including progerias, cardiomyopathies, and muscular dystrophies [22,68,204,205,206,207,208]. Much has been learned about the functions of these proteins by studying their deletion in developmental and differentiation models. The well-studied Lamin A/C deletion mouse model [72] (later shown to result in the expression of a C-terminally truncated Lamin A lacking in key functional domains, and now referred to as the Lmna^Δ8–11/Δ8–11^ mouse [209]) leads to the birth of apparently normal mice that develop severe muscular dystrophy by 8 weeks [72], mirroring the impact of Lamin A mutations in human disease. Nuclei from Lmna^Δ8–11/Δ8–11^ MEFs are frequently crescent-shaped (lobulated) and have reduced size, compared to the rounded nuclei of wild-type MEFs [72,210]. Immunostaining demonstrated the presence of a microtubule organizing center (MTOC) at the central fold of these nuclei. Treatment with the microtubule inhibitors, nocodazole or taxol, increased the circularity of Lmna^Δ8–11/Δ8–11^ MEFs, demonstrating a role of microtubules in maintaining the abnormal shape of Lmna^Δ8–11/Δ8–11^ MEFs [210]. Another example using cells from the Lmna^Δ8–11/Δ8–11^ mouse studied the differentiation of murine embryonic stem (ES) cells. Undifferentiated wild-type ES cells have a flattened ovate shape and differentiate into primitive endoderm cells with more spherical nuclei and increased mRNA levels of Lamin A/C and Emerin upon treatment with retinoic acid [211]. Nuclei of Lmna^Δ8–11/Δ8–11^ or Emerin knockout (KO) ES cells are not significantly different in shape from those of wild-type ES cells, but retinoid-treated Lmna^Δ8–11/Δ8–11^ or Emerin KO cells retain a flattened appearance, indicating that Lamin A/C and Emerin are needed for the change in nuclear shape that occurs during ES cell differentiation [211]. Lmna^Δ8–11/Δ8–11^ and Emerin deletion also inhibited the alterations in gene expression associated with the differentiation of ES cells, demonstrating a link between nuclear shape change and gene expression [211].

In the Lamin B1-deficient (Lamin B1^Δ/^ ^Δ^) mouse model, an insertional mutation in the Lamin B1 gene encodes a truncated Lamin B1 fusion protein lacking the C-terminal half of Lamin B1 [75]. Lamin B1^Δ/^ ^Δ^ mice develop with bone and lung defects and die upon birth [75]. MEFs from Lamin B1^Δ/^ ^Δ^ mice have malformed nuclei with blebs and lobules, and increased ploidy, indicating improper cell division [75]. Brain development is abnormal in Lamin B1^Δ/^ ^Δ^, with many neuron cell nuclei exhibiting a single bleb, unlike wild-type neurons, which rarely have blebs [75]. Lamin B2 knockout (^-/-^) mice also have abnormal brain development and die shortly after birth [212]. Lamin B2 ^-/-^ neurons do not have blebs but are atypically elongated [75]. These studies show the importance of B-type lamins for brain development and suggest that the absence of human diseases stemming from B-type lamins results from the embryonic lethality of such a condition.

Table 1 lists a number of proteins whose mutation, depletion, or overexpression have been reported to cause alterations in nuclear morphology. In addition to lamins [68,69,73,77], lamin-associated proteins [66,67,86], and LINC complex components [91,94,97,98], other nuclear proteins, such as epigenetic enzymes [100,101,103,104], histone variants [106,107], nucleosome binding proteins [108], and nuclear pore-related proteins [109,110,112,113], have been demonstrated to regulate nuclear morphology. In addition, the depletion of certain cytoskeletal-associated proteins [80,114,115,116,117,118], the transcription factor GATA6 [119], the ER-localized LEM protein, ANKLE2 [63], and the SPANX cancer/testis antigen [122] each led to altered nuclear appearance. This list is not meant to be all-inclusive and other publications have identified additional proteins that regulate nuclear morphology [109,213,214]. While many proteins in Table 1 directly impact the structure of the nuclear envelope or lamina (e.g., lamins, LEM proteins), several of the listed proteins affect nuclear morphology indirectly. For example, mutation of ZMPSTE24 inhibits Lamin A processing [215,216], loss of GATA6 or DIAPH3 decreases Emerin levels [80,119], and silencing of SIRT2 reduces the deacetylation of ANKLE2 [65].

Some of the examples in Table 1 are notable because they illustrate the coordination of changes in nuclear morphology with alterations in chromatin organization. For example, mH2A (macroH2A1 and macroH2A2) histone variants are important regulators of gene expression [217]. mH2As have been shown to have decreased expression in many malignancies, including breast, lung, and liver cancer and melanoma [217,218], although, thus far, no links between mH2A and pancreatic cancer have been reported [219]. In most experimental studies, mH2A1 and mH2A1 act as tumor suppressors, with their loss of expression correlating with increased tumor cell growth and reduced survival, although, in a few cases, mH2A expression promotes cancer cell growth [217,218,220,221]. Douet et al. [106] stably knocked down mH2A1 and mH2A2 in HepG2 hepatic carcinoma cells. As noted in Table 1, a reduction in mH2As led to nuclear alterations, including increased size and presence of lobulations and blebs. mH2A depletion also caused an overall loss of heterochromatin, especially at the nuclear periphery and around nucleoli. ChIP-seq studies of mH2A2 genomic distribution showed that in nondepleted cells, mH2A2 peaks were in general inversely correlated with gene transcription, suggesting that mH2A is associated with heterochromatin. Further, mH2A2 and H3K9me3 comarked a number of DNA repeat regions, and mH2A1/2 knockdown caused a diffusion in the localization of repeat regions in the nucleus and an increase in transcription from some DNA repeat regions. Proximity ligation assays showed that mH2As interact with Lamin B1 and H3K9me3 at the nuclear lamina. Additionally, mH2A2 ChIP-seq peaks were shown to overlap with LAD regions from human IMR90 cells. Finally, knockdown of mH2A1/2 led to a dissociation of Lamin B1 from repeat regions, as assessed by Lamin B1 ChIP-seq. These data support previous work that demonstrated that mH2A1 is associated with and involved in the organization of LADs [107]. Together, these results suggest that mH2As play a role in higher levels of chromatin organization by anchoring repeat regions to the nuclear lamina. Similarly, HMGN5 is a high-mobility group protein that binds to nucleosomes, competing with linker histones for nucleosome occupancy. While the linker histones promote and maintain chromatin condensation, HMGN5 expression leads to chromatin decompaction [222]. Overexpression of HMGN5 alters the expression of ~2000 genes, alters the distribution of heterochromatin [223], and causes nuclear blebs [108], thus providing an example of altered chromatin organization in association with aberrant nuclear morphology.

Several of the listed proteins in Table 1 demonstrate how the actin cytoskeleton can affect nuclear function. PPP1CB and PPP1R12A are subunits of the myosin phosphatase complex that oppose actin/myosin contractility. Depletion of either of these subunits in HeLa cells was shown to cause extensive nuclear blebs, loss of nuclear circularity, loss of continuity of the nuclear lamina, and nuclear rupture [118]. Nuclear morphology was restored by the treatment of cells with the myosin inhibitor, blebbistatin, the ROCK inhibitor, Y-27632, and the rho inhibitor, c3 toxin, supporting the involvement of actomyosin activation in nuclear disruption in PPP1CB- and PPP1R12A-depleted cells. Imaging studies led to the observation that actin bundles were compressing regions of nuclei in PPP1CB- and PPP1R12A-depleted cells, leading to nuclear aberrations. Thus, forces generated by actin/myosin activity outside the nuclei were responsible for nuclear deformation in this case.

Although our literature survey for Table 1 did not identify microtubule-associated proteins that regulate nuclear shape, a number of studies have demonstrated the importance of the microtubule cytoskeleton for the regulation of nuclear shape. The importance of microtubules for maintaining the abnormal shape of Lmna^Δ8–11/Δ8–11^ MEFs [210] was discussed above. Biedzinski et al. [224] studied the differentiation of hematopoietic stem and pluripotent cells (HSPCs) into myeloid progenitor cells (MPCs). Nuclei of HSPCs are small and spherical, while MPCs are twice as large and deformed, with one or more large invagination [224]. In MPCs, the MTOC was shown to be closely associated with nuclear invaginations [224]. Short-term treatment (3h) of MPCs with microtubule inhibitors did not reduce nuclear deformation. However, 48-72 h treatment of HSPC with the microtubule inhibitor, taxol, or the dynein inhibitor, ciliobrevin, during differentiation to MPCs prevented the deformation of myeloid cell nuclei but not the increase in size. In this study, total lamins were apparently unchanged, although the distribution of Lamin B was altered [224]. In another example, the nuclei of cardiomyoctes depleted of the intermediate filament, Desmin, or depleted of Nesprin-3 were shown to decrease in size and develop extensive narrow invaginations [225]. Microtubules were observed to be present in these invaginated regions. Treatment of cells with microtubule inhibitors before the development of nuclear abnormalities (24 h after shRNA treatment) prevented the formation of nuclear invaginations [225]. However, treatment with microtubule inhibitors after nuclear infolds had formed did not reverse these nuclear abnormalities. Lamin A/C and Lamin B1 levels were not altered by Desmin depletion; however, this treatment greatly altered gene expression and reduced the interaction between Lamin B and chromatin, as judged by Lamin B1 ChIP-seq assays [225]. Together, these studies demonstrate the involvement of microtubules in nuclear deformation and provide examples of nuclear shape alterations occurring in the absence of gross changes in lamin levels.

## 4. Alterations in Nuclear Structure and Chromatin Organization in Pancreatic Cancer

We have noted that alterations in expression or mutations of individual proteins can result in changes in nuclear morphology and/or chromatin organization. However, the reality in cancer is that multiple genomic, transcriptomic, and epigenomic aberrations are present simultaneously and could additively impact nuclear and chromatin structure. Below, we describe what is known about chromatin domains in PDAC, mention recent studies of individual proteins whose nuclear alterations impact pancreatic cancer, and summarize new work enhancing our global view of altered nuclear dynamics in pancreatic cancer subtypes.

To our knowledge, few studies have investigated chromatin compartmentation at any level in PDAC. Timme et al. investigated the radial positioning of chromosome 8 (CT8) in the normal human non-neoplastic pancreatic ductal epithelium vs. PDAC [226]. The authors noted no significant changes in chromosome territory 8 (CT8) radial positioning or volume between the groups, but they did identify that CT8 was shaped irregularly in PDAC. No LAD studies have been performed in pancreatic cancer models. McDonald et al. investigated the epigenomic landscape alterations in PDAC with regional (peritoneal) vs. distant (liver or lung) metastases [185]. The authors observed a global reduction in H3K9me2 and H3K9me3 in samples from distant vs. peritoneal metastases. Primary tumors from patients with distant metastases showed similar reductions in H3K9 methylation. Further, peritoneal metastases displayed greater H3K9me2 in large regions of H3K9me2 (LOCKs) compared to distant metastases and their matched primary tumor subclones. DNA methylation was decreased within these LOCKs in the peritoneal samples. Additional gene expression studies identified 6-phosphogluconate dehydrogenase (PGD) of the oxidative branch of the pentose phosphate pathway as playing a role in the greater invasiveness of the distant vs. regional metastases, and demonstrated that PGD inhibition reversed the depletion of H3K9me2 at LOCKs and decreased the tumorigenic properties of cells from distant metastases. These studies suggest that PGD inhibitors may be beneficial in PDAC.

Only a few studies have investigated nuclear lamina proteins in pancreatic cancer. For example, Lamin B1 was reported to overexpressed in pancreatic cancer vs. normal pancreas, and high Lamin B1 expression was associated with less differentiated tumors and shorter patient survival [227]. Further, in this study, depletion of Lamin B1 decreased pancreatic cancer cell growth in vitro and in xenografts in mice [227]. Similarly, LAP2 is overexpressed in pancreatic cancer, and the proliferation and migration of PANC1 PDAC cells was inhibited by LAP2β reduction [228].

Some of the proteins noted in Table 1 as affecting nuclear morphology (BRG1, KPNA7, GATA6) are highly relevant to PDAC. Depletion of BRG1 (SMARCA4) leads to altered nuclear morphology, including irregular nuclear shape and the presence of nuclear bulges and septa [100]. In addition, expression of an ATPase-deficient mutant of BRG1 increases nuclear size [101]. BRG1 is a component of the SWItch/sucrose non-fermentable (SWI/SNF) chromatin remodeling complex. Chromatin remodelers modulate transcription by affecting nucleosome structure and positioning, and loss of BRG1 and other SWI/SNF components lead to large-scale changes in chromatin accessibility and gene expression [229,230]. BRG1 has been shown to be mutated or silenced in up to 10% of human pancreatic cancer patients [231,232,233]. In mouse models, the absence of BRG1 was found to increase the formation of intraductal papillary mucinous neoplasms from pancreatic ductal cells but decrease the development of PDAC from acinar cells [234]. A recent study demonstrated that in pPtf1a-Cre^ER^; Kras^G12D^ mice, loss of BRG1 inhibited the formation of PanINs and decreased acinar to ductal metaplasia via downregulation of SOX9 [235]. Thus, loss of BRG1 function in chromatin organization led to changes in cell fate relevant to PDAC development. Another protein that impacts nuclear shape is KPNA7, a newly identified importin [110]. KPNA7 depletion results in extensive nuclear lobulation and loss of circularity in pancreatic cancer cells [110]. The *KPNA7* gene occurs within the 7q21-q22 amplicon, a region frequently amplified in pancreatic cancer [236]. KPNA7 is not expressed in most adult tissues but is expressed in some human pancreatic cancers and pancreatic cancer cells [236,237]. Depletion of KPNA7 in pancreatic cancer cells led to reduced proliferation rates and defects in mitosis [110,237]. The involvement of the transcription factor, GATA6, in PDAC is discussed below.

Results from a number of investigational studies have led to a convergent view that most pancreatic adenocarcinomas can be classified into two subtypes (classical and basal-like (also called squamous)) based on transcriptional profiles, with the basal-like group having a worse prognosis [232,238,239,240,241,242]. Lomberk et al. performed genome-wide analysis using RNA-seq, DNA methylation, and ChIP-seq for repressive and activating histone marks, and demonstrated that the two different PDAC subtypes could be distinguished by histone mark and DNA methylation patterns [240]. We recently examined the differences in chromatin organization between classical PDAC and adenosquamous carcinoma of the pancreas (ASCP), a basal-like pancreatic cancer with a histologically squamous phenotype, using ATAC-seq (Assay for Transposase-Accessible Chromatin using sequencing) analyses [243]. We found that ASCP had a distinct pattern of open chromatin compared to classical PDAC [243]. In particular, open chromatin peaks associated with the SMYD2 and RORC loci were higher in ASCP than classical PDAC [243]. Together, these studies support the concept that classical and basal-like pancreatic cancers differ in their overall chromatin organization.

A recent paper used fluorescence-activated cell sorting (FACS) to enrich for epithelial cells from normal pancreas and pancreatic cancer samples and then performed genome-wide DNA methylation analysis and RNA-seq [244]. Consistent with previous studies [245,246,247], PDAC tumors were globally DNA hypomethylated, with regions of hypermethylation associated with CpG islands near genes. Most losses in DNA methylation occurred in nongenic regions not associated with CpG islands. Two distinct PDAC groups were identified based on their DNA methylome patterns. One group, with lower overall DNA methylation, had gene expression signatures associated with the basal-like PDAC subtype, whereas the other group corresponded to the classical PDAC subtype transcriptional profile. Most of the differentially DNA methylated regions between the two subtypes were associated with repeated element regions of the genome, with long interspersed nuclear elements (LINEs), short interspersed nuclear elements (SINEs), and endogenous retroviruses (ERVs) being less DNA methylated in the basal-like group. Further, transcripts arising from LINEs and ERVs were more highly expressed in the basal-like group than in the classical group. This study further found an that interferon signature was associated with the basal-type group and suggested that treatment of basal-like subtype patients might benefit from treatments via the JAK/STAT pathway to inhibit interferon signaling.

Finally, GATA6, a transcription factor important for differentiation in the pancreas [248], has been shown to be expressed and sometimes amplified in the classical PDAC subtype but is absent in basal-like PDAC [232,238,240,249]. As noted earlier, GATA6 depletion is sufficient to cause nuclear aberrations. Recent studies have increased our understanding of the mechanisms of silencing of GATA6 in PDAC. First, GATA6 was shown to be repressed by EZH2, the primary chromatin enzyme that dimethylates and trimethylates H3K27 [250]. EZH2 was shown to be expressed in human PDAC and its expression was negatively correlated with GATA6 expression in these tissues. Second, genome-wide studies of 5-methylcytosine (5mC) and 5-hydroxymethylcytosine (5hmC) on DNA in basal-like vs. classical PDAC samples revealed that 5hmC was globally lower in basal-like samples [251]. Hydroxylation of 5mC to 5hmC by TET 1-3 enzymes is thought to be a step toward DNA demethylation [252]; thus, sites without 5hmC may be more DNA methylated and thus more repressed. Further investigations in PDAC cells demonstrated that 5hmC levels were governed by TET2 levels, which varied between cell lines, and that TET2 expression was correlated with GATA6 expression among cell lines. The authors concluded that 5mC marks on DNA were higher on the GATA6 promoter in basal-like cells as a result of low TET2 activity, leading to GATA silencing. Based on its findings, this report suggests that metformin and ascorbic acid might be used to stimulate TET2 activity to revert basal-like tumors to less unfavorable classical PDAC. Together, the above studies demonstrate how investigations of chromatin organization of PDAC subtypes are leading to a new understanding of this disease and providing leads towards new, personalized therapeutic approaches for PDAC.

## 5. Unanswered Questions and Future Directions

Investigations into aberrant nuclear structure due to protein alterations have not fully identified the mechanisms by which nuclear changes take place in cancer cells. We noted one study where the cytoskeleton appeared to deform the nucleus by exerting force from outside the nucleus [118], whereas other reports showed that abnormal nuclear shape was independent of the cytoskeleton [100,115]. In some cases, loss of the integrity of the nuclear lamina or nuclear envelope has been mentioned as a possible mechanism for blebs or lobulations arising from the nucleus [72,110,112,115,253]. One study removed nuclei from cancer cells with lobulated nuclei and found that the aberrant nuclear shape was retained in the isolated nuclei, suggesting that an abnormal nuclear shape can be inherent to the nucleus and not require outside forces [254]. Finally, the altered nuclear shape in cancer cells could be a result of misfolded chromatin [255] or aneuploidy [256]. Most likely, multiple mechanisms are responsible for the altered nuclear morphology in cancer cell nuclei. Many questions remain concerning the origins of nuclear deformation and its interaction with chromatin organization in pancreatic cancer.

What are the roles of nuclear lamina-associated proteins in altered nuclear morphology in pancreatic cancer cells? The mechanisms by which individual nuclear lamina proteins regulate nuclear shape remain only partially understood. Many proteins of the nuclear lamina (e.g., Emerin, LEMD2, LAP1, ANKLE2) play roles during or at the end of mitosis, in addition to their roles in heterochromatin regulation during interphase [43,63,257,258]. A study in Xenopus egg extract nuclei as well as mammalian cells suggested that nuclear size is proportional to the amount of total lamin (either A-type or B-type) available, with a higher concentration threshold after which lamins decrease nuclear size [78,213], leading to the concept that lamin expression is correlated with nuclear size [259]. However, it is difficult to reconcile this model with the complex nuclear dysregulation that occurs in cancer cells. For example, breast cancer cells are reported to have lower Lamin A/C than normal mammary epithelial cells [260,261,262,263], but typically have much larger nuclei than normal cells [264,265,266]. In addition, both Emerin and Lamin A loss (see Table 1) are reported to reduce nuclear size in some cases but increase nuclear size in others [73,74,78,79,81,82,267]. Perhaps these questions can be resolved by studying the role of nuclear lamina proteins within the context of the intracellular machinery present within different cell models, such as the relative stoichiometry of interacting proteins.

Do alterations in nuclear lamina-associated proteins contribute to nuclear deformation in pancreatic cancer cells? Very little has been reported concerning the expression levels or functional roles of nuclear membrane proteins in pancreatic cancer. We mentioned above the observations that Lamin B1 is overexpressed in pancreatic cancer and high Lamin B1 expression is associated with poorly differentiated tumors and shorter patient survival [227], and that LAP2 is overexpressed in pancreatic cancer, and that LAP2β depletion decreases PDAC cell growth [227]. However, no studies have been performed to determine if Lamin A/C, Emerin or other nuclear lamina proteins are altered in expression or localization in PDAC, or if these proteins play roles in altered nuclear morphology in PDAC. Further, if alterations in nuclear lamina proteins do occur, how is this accomplished? For example, are alterations in protein expression driven by oncogenic signaling or changes in degradation rate?

**Figure 3:**
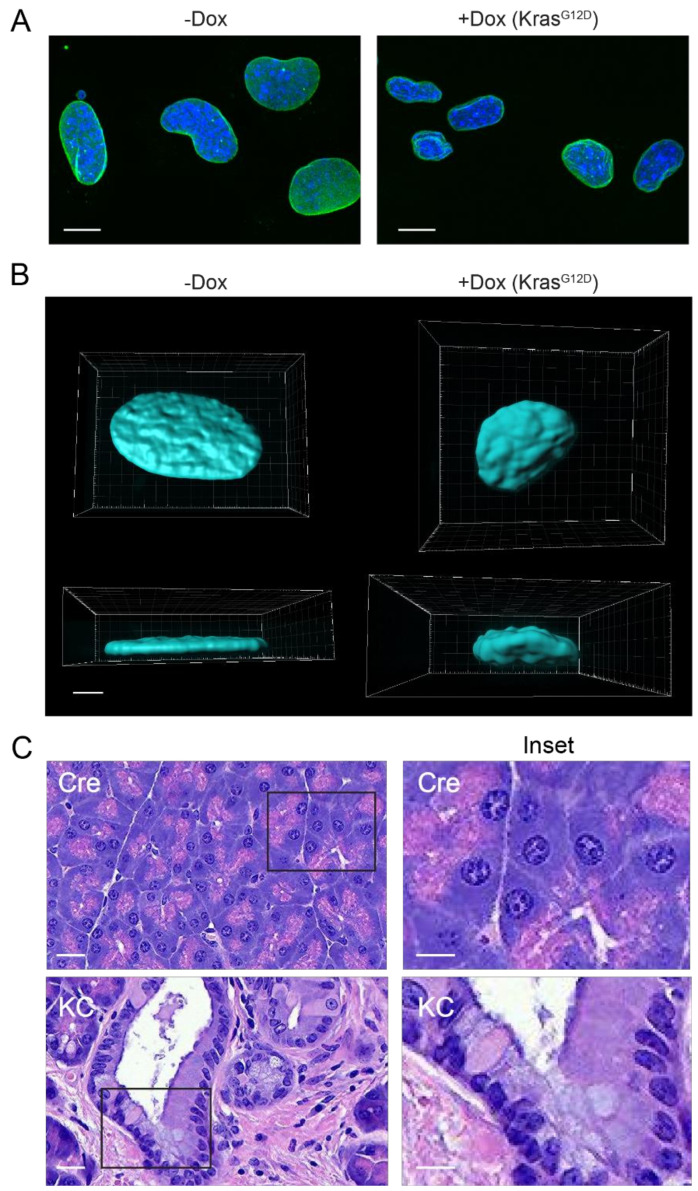
Oncogenic KRAS drives aberrant nuclear morphology in vitro and in vivo. (**A**) 4292F murine PDAC cells with a doxycycline (Dox)-inducible KRAS^G12D^ are shown, lamin B1 (green), DAPI (blue). Cells that are not expressing oncogenic KRAS (-Dox) present large and rather uniform nuclei. Induction of oncogenic KRAS with Dox causes a significant reduction in nuclear size and presents lamina alterations. Bar = 10 µm. (**B**) A 3D reconstruction from Z-stacks of DAPI signal from 4292F PDAC cells grown without or with Dox. All images were sized proportionally. (**C**) Murine H&E tissue sections from normal pancreas of Cre mice and PDAC pancreas from KRAS^G12D^ mice (KC). Normal pancreas displays larger and more uniform nuclei than those from PDAC. Bar = 100 µm. Inset regions are indicated by black rectangles and shown at higher magnification at right. Inset bar = 50 µm.

Does altered nuclear morphology or its underlying mechanisms contribute to transformation and oncogenicity in PDAC? Changes in nuclear lamina proteins could lead to the reorganization of chromatin (e.g., LADs), which fosters large-scale alterations in gene expression that promote cancer [268,269]. The clearest example of this potential mechanism is the loss of nuclear-periphery-associated heterochromatin and changes in gene expression observed upon mutation or depletion of lamins in various cell types [270,271,272,273,274,275,276]. However, despite extensive research demonstrating that alterations in individual proteins can change nuclear structure and chromatin organization, it is still unknown if such modulations drive oncogenic processes or simply are the inevitable result of multiple changes acquired by cancer cells as they evolve and survive. Our own group seeks to investigate this question using a reductive model for cell transformation. Utilizing inducible KRAS^G12D^ mouse pancreatic cancer cell lines, we find that the overexpression of oncogenic mouse KRAS^G12D^ decreases the nuclear area in these cells (Figure 3A). The 3D reconstruction of cellular images shows that cells with KRAS^G12D^ expression are not only smaller in volume but also altered to a spherical shape, unlike cells without KRAS^G12D^, which are more flattened (Figure 3B). We also compared the size of nuclei in the normal mouse pancreas vs. in pancreatic tumors from mice with a Cre-driven conditional pancreatic expression of oncogenic mutant KRAS*^G12D^* (KC mice) and found that tumor nuclei were smaller than normal mouse exocrine nuclei (Figure 3C). These findings indicate that turning on oncogenic signaling is sufficient to alter nuclear morphology. We will use this model to perform genome-wide studies to determine how KRAS decreases nuclear size and impacts chromatin organization, and how these factors impact the proliferative state of these cells.

Alternatively, dysregulation of nuclear lamina-associated proteins has the potential to increase nuclear ruptures, cause DNA damage, or affect the fidelity of mitosis [52,132,277,278,279,280,281,282]. Lamin A (or A/C) is reported to be decreased in breast, gastric, and ovarian cancer, with low Lamin A levels associated with shorter patient survival [261,263,267,283,284]. Such observations have led to the hypothesis that a weakened nuclear lamina (e.g., by decreased Lamin A) in cancer cells leads to genomic instability (e.g., aneuploidy) that bestows cancer cells with genomic heterogeneity, which could contribute to genomic evolution during oncogenesis [119,256,267,285,286]. In contrast, Lamin A is increased in hepatocellular carcinoma and invasive prostate cancer, and acts as a positive regulator of cell growth and migration in these cancer cell types [287,288], illustrating that Lamin A/C loss is not a universal feature of cancer. Although decreased Lamin A/C has been linked with softer nuclei and increased migration or invasiveness of cancer cells [189,289], a study comparing four PDAC cell lines found that invasiveness was positively correlated with increased stiffness and Lamin A levels among these cells [190]. Clearly, much more needs to be learned about the interrelationships between nuclear morphology, gene expression, nuclear stiffness, and genetic instability in pancreatic cancer.

Is the karyoplasmic ratio altered and of significance in pancreatic cancer? A striking cytological feature of cancer cells is the frequently observed change in the karyoplasmic ratio (nuclear:cytoplasmic ratio). The karyoplasmic ratio is one of the prominent features used by histopathologists for cancer diagnosis and prognosis [290]. In cancers, the nuclear:cytoplasmic ratio typically increases; however, the functional aspect of this feature is not well-understood. Further, nuclear size regulation has been shown to be independent of cell size [291], which suggests that nuclear size control is not necessarily a passive secondary effect of cell activity. Further investigation will be necessary to determine if the karyoplasmic ratio is altered in different subtypes (e.g., classical vs. basal-like, moderately vs. poorly differentiated) of pancreatic cancer.

Chromatin organization has been studied at many levels using increasingly sophisticated tools and analyses. In reviewing the literature, it has become clear that chromatin domains such as constitutive heterochromatin, LADs, LOCKs, and global DNA hypomethylation patterns are linked to the distribution of repetitive elements of the genome. It has also been separately reported that pancreatic cancer cells aberrantly express repeat element transcripts [292]. Earlier studies may have underestimated the connections between repeat elements and epigenomics due to difficulties in annotating repeat sequences [292]. However, recent techniques have largely overcome these difficulties. It has been suggested that repeat elements may be major structural components involved in high-order chromatin folding [160,293,294,295]. Hopefully, future studies will utilize multi-omic approaches to explore this idea in the context of cancer.

In conclusion, studies of the mechanisms underlying altered nuclear morphology and chromatin organization in PDAC are few in number at present, but are already presenting clues to the pathology of cancer cells, identifying large-scale epigenetic differences between PDAC subtypes, and providing leads to new therapeutic approaches for pancreatic cancers and other malignancies.

## Figures and Tables

**Table 1:** Protein alterations affecting nuclear morphology.

Protein Alteration	Nuclear Changes	References
Lamins and Associated Proteins		
Lamin A mutation	Lobulations	[68,69,70,71]
Lamin A truncation	Increased area, blebs, lobulations, aneuploidy	[72,73,74]
Lamin B1 truncation	Blebs	[74,75,76]
Lamin B2 deletion	Elongation	[76]
Lamin B2 deletion	Ruptures	[77]
Lamin A, B1 or B2 depletion	Decreased area	[78]
Lamin A, B1 or B2 overexpression	Increased area	[78]
Emerin mutation	Increased area	[79]
Emerin deletion	Increased area	[73]
Emerin depletion	Increased area, lobulations, blebs	[79,80]
Emerin depletion	Reduced area, invaginations	[81]
Emerin overexpression	Increased nuclear area	[82]
LEM2 depletion	Lobulations	[66]
LAP1 mutation	cytoplasmic channels, lobulations, invaginations	[83,84]
LAP1 deletion	Ruffled	[85]
LAP1 overexpression	Lobulations	[86]
LAP1C overexpression	Invaginations	[87]
LAP2b depletion	Increased area, hyperploidy	[67]
ANKLE2 depletion	Lobulations, increased area, hyperploidy	[55,63,65]
ZMPSTE24 mutation	Lobulations	[88,89]
**LINC Complex Proteins**		
Nesprin 1 mutation	Lobulations	[90]
Nesprin 1 or Nesprin 2 depletion	Lobulations, increased area	[91,92]
SUN1 mutation	Enhance blebs in Lamin A mutant cells	[93]
SUN1/SUN2 depletion	Lobulations	[94,95]
SUN2 overexpression	Lobulations	[96]
Torsin deletion	Intraluminal blebs	[97,98]
Torsin 1 overexpression	Blebs, invaginations	[99]
**Chromatin Enzymes**		
BRG1 depletion	Lobulations	[100]
BRG1-ATPase deficient	Increased area	[101]
ARID1A	Increased area	[102]
RING1B depletion	Increased area, hyperploidy	[103]
MOF deletion	Blebs, micronuclei	[104]
NCAPH2 or NCAPD3 depletion	Lobulations, increased area	[105]
SMC2 depletion	Lobulations	[105]
**Nucleosome Proteins**		
mH2A1 and mH2A2 deletion	Lobulations, blebs, increased area	[106,107]
HMGN5 overexpression	Blebs	[108]
**Nuclear Pore-Related Proteins**		
ELYS depletion	Decreased size	[109]
KPNA7 depletion	Lobulations	[110]
NUP53 depletion	Lobulations	[111]
NUP98 depletion	Lobulations	[111,112]
NUP153 depletion	Lobulations, invaginations	[113]
**Cytoskeletal-Associated Proteins**		
Cofilin and ADF depletion	Lobulations	[114]
DIAPH3 depletion	Lobulations	[80]
a-dystrobrevin depletion	Lobulations, blebs, septa	[115]
EPB41 depletion	Blebs, lobulations	[116]
LLGL1 or LLGL2 depletion	Increased area	[117]
PPP1R12A or PPP1CB depletion	Lobulations, blebs	[118]
**Other**		
GATA6 decrease	Larger size, lobulations, aneuploidy	[119]
NOP53 depletion	Lobulations	[120]
Nucleophosmin depletion	Lobulations	[121]
SIRT2 depletion	Increased area	[65]
SPANX depletion	Increased area, lobulations	[122]
STIP1 depletion	Reduced size, invaginations	[81]
TMEM170A depletion	Increased area, lobulations	[123]
YBX1 depletion	Lobulations	[124]

## Data Availability

Not applicable.
